# Age and gender-specific distribution of metabolic syndrome components in East China: role of hypertriglyceridemia in the SPECT-China study

**DOI:** 10.1186/s12944-018-0747-z

**Published:** 2018-04-20

**Authors:** Boren Jiang, Yanjun Zheng, Yingchao Chen, Yi Chen, Qin Li, Chunfang Zhu, Ningjian Wang, Bing Han, Hualing Zhai, Dongping Lin, Yingli Lu

**Affiliations:** Department of Endocrinology and Metabolism, Shanghai Ninth People’s Hospital, Shanghai JiaoTong University, School of Medicine, Shanghai, 200011 China

**Keywords:** Metabolic syndrome, Hypertriglyceridemia, Cross-sectional design, Gender, Chinese

## Abstract

**Background:**

Chinese population are experiencing remarkably changes of economic and cultural environments. The present study was to examine the prevalence of metabolic syndrome (MetS) by age between genders and to investigate the current characteristics of MetS and its components in China.

**Methods:**

SPECT-China is a population-based cross-sectional survey on Chinese adults aged ≥18 years in East China. A total of 10,441 Chinese residents participated in anthropometric and laboratory measurements. Of these, 9969 subjects (females, 5868) were eligible for the data analysis reported here. Estimates of the prevalence of MetS and its components were calculated. Presence of MetS was defined based on the IDF/AHA harmonized criteria. MetS z-score was calculated to evaluate the degree of total metabolic disorder.

**Results:**

The age-standardized prevalence of the metabolic syndrome was 22.0% (21.9% in men and 22.0% in women). Unlike the continuous MetS rise with age in females, the MetS prevalence in males remained stable among 46–55, 56–65 and > 65 yrs. age groups (31.2%, 31.4%, 32.5%, *p* = 0.538). In the five components of MetS, contrary to the elevated BP and BG disorders, the frequency of TG disorders decreased with age in males (46.6%(46–55 yrs), 37.2% (56–65 yrs), 27.7%(> 65 yrs), *p* < 0.001). Multivariable logistic regression showed that in males, more TG disorders were associated with higher BMI, higher educational level and current nonsmoker. In the MetS subjects, the 3-factor combinations which included TG disorders decreased with age in both genders. The whole metabolic profile became better in older male MetS subjects, which was opposite to the female.

**Conclusion:**

Our results showed a distinct age-related prevalence of MetS between genders in dramatically changed China, in which the TG disorders played an important role. More targeted measures need to be taken to meet the serious challenges of metabolic diseases.

**Trial registration:**

ChiCTR-ECS-14005052, Survey on Prevalence in East China for Metabolic Diseases and Risk Factors (SPECT-China).

**Electronic supplementary material:**

The online version of this article (10.1186/s12944-018-0747-z) contains supplementary material, which is available to authorized users.

## Background

Metabolic syndrome (MetS) is defined as a cluster of metabolic abnormalities including central obesity, hypertension, high plasma triglycerides, decreased high density lipoprotein (HDL) cholesterol and glucose intolerance. Having any three of the five abnormal indicators above has been defined as MetS [1]. It has been shown that MetS increases the risk for atherosclerotic cardiovascular disease (ASCVD) and diabetes mellitus [2, 3], which are major contributors to morbidity and mortality all over the world [4–6], including China [7].

Epidemiologic studies have demonstrated differences in MetS prevalence by geography, age, gender and ethnicity. Taking age as an example, from NHANES III in the US [8], a marked increase in prevalence of the MetS from 20 years of age through the sixth decade of life was noted for men and through the seventh decade for women. However, prevalence declined in the eighth decade of life. A different pattern was observed from the Norwegian HUNT II Study [9]. In this study, the MetS prevalence increased linearly with age into the ninth decade of life. The MetS prevalence and components also markedly varied with aging in the same group of population. From 1988 to 1994 to 2007–2012, the prevalence of MetS increased from 25.3% to 34.2% in the US [10]. Our previous data showed the unstandardized prevalence of MetS elevated from 29.65% (2005) to 45.49% (2014) from a community study in China [11]. Although the underlying causes of these variations were not clearly demonstrated yet, they usually have been attributed to differences in genetic and environmental factors such as dietary patterns, activity, stress levels and socioeconomic status [12, 13].

Chinese people are experiencing remarkably changes of economic and cultural environments, such as factors related to rapid nutritional transition, rural-to-urban migration, adoption of a sedentary lifestyle and mental stress [14, 15], which may contribute to the progression of metabolic syndrome. It is crucial to get undated knowledge about the characteristics of metabolic syndrome in China, which can help design specific preventative and therapeutic strategies. Several previous epidemiologic studies have displayed important information about the national or regional prevalence of MetS in China [16–20]. However, detailed data about the prevalence and characteristics of MetS by age in different genders are scarce.

The present study was to examine the prevalence of MetS by age between genders and to investigate the current characteristics of MetS and its components in China. We aimed to get a better understanding of MetS for future specific prevention and treatment in China.

## Methods

### Study design and population

SPECT-China (ChiCTR-ECS-14005052, www.chictr.org.cn) is a population-based cross-sectional survey on prevalence of metabolic diseases and risk factors in East China, which is made up of Shanghai and 7 provinces with a population of approximately 395 million in 2011, accounting for 29.2% of people in China. Detailed sampling information was described in a previous study [21]. In brief, the study was conducted from February 2014 to December 2015. Twenty-two residential sites in Shanghai, Zhejiang, Jiangxi, Jiangsu and Anhui Province were selected using a stratified and cluster sampling method. Chinese citizens ≥18 years old who had lived in their current area for ≥6 months were selected. We excluded subjected with severe communication problems, acute illness or who were unwilling to participate. A total of 10,441 subjects were enrolled in SPECT-China study finally. This study was conducted according to the principles laid down in the Declaration of Helsinki of 1975, was approved by the Ethics Committee of Shanghai Ninth People’s Hospital, Shanghai JiaoTong University School of Medicine. All patients to be included signed the informed consent. For participants who were illiterate, we obtained written informed consent from their proxies. After excluding individuals who had missing MetS components (WC, BP, FBG, TG or HDL) data or were pregnant or had active malignant tumors (*n* = 472), a total of 9969 subjects (females, 5868) were finally analyzed.

### Demographic, anthropometric and laboratory measurements

Subjects were interviewed face-to-face to complete pre-tested questionnaires covering sociodemographic information, medical history of coronary heart diseases, diabetes, hypertension and lipid disorders.

Marital status was recorded in four categories: single, married/co-habiting, divorced, and widowed. Education level, determined on the basis of the highest level achieved, was classified into three categories: primary school or below, middle school, high school or above. Occupational position was categorized as non-manual, manual and self-employed. Cigarette smoking was assessed by self-report from the question “Do you smoke cigarettes now?” (yes/no). Alcohol consumption was assessed based on how often participants consumed wine, beer, or hard liquor, as follows: less or equal than one drinks per week (none current drinker), and two drinks or more per week (current drinker).

Height and weight were measured to the nearest 0.5 cm and 0.1 kg, respectively, with the participants wearing light-weight clothing and without shoes. Body mass index (BMI) was calculated as weight (kg) divided by height squared (m2). WC was measured on standing participants midway between the lower edge of the costal arch and the upper edge of the iliac crest using a non-elastic tape (to the nearest 0.5 cm). All anthropometric measurements were taken in duplicate and the averages of these measurements were used in the analyses.

Resting systolic and diastolic BP was measured three times at 1-min intervals using a standard mercury sphygmomanometer after a 5-min rest. The average of the second and the third readings were used in the analyses.

Fasting venous blood samples were collected in the morning after at least 8 h of fasting. The samples for plasma glucose test were collected into vacuum tubes with anticoagulant sodium fluoride and centrifuged on the spot in 1 h after collection. Blood samples were shipped in dry ice to a central laboratory within 2–4 h of collection. FPG, HDL-C and TG were analyzed enzymatically using an autoanalyzer. All laboratory equipment was calibrated, and blinded duplicate samples were used for these analyses.

### Definition of metabolic syndrome

MetS was defined based on the IDF/AHA harmonized criteria [1]. Thus, positive diagnosis of the syndrome was established when at least three of the following were present: (1) Waist circumference ≥ 90 cm in men or ≥ 80 cm in women [22]; (2) HDL cholesterol < 1.0 mmol/L in men or < 1.3 mmol/L in women; (3) Serum TGs ≥1.7 mmol/L (4) Serum glucose level ≥ 5.6 mmol/L; (5) Blood pressure ≥ 130/85 mmHg. Treatment with anti-hypertensive, hypoglycemic or lipid-lowering drugs was considered as alternate indicators of the latter three components. Age-standardized incidence rates of MetS were calculated using direct standardization with population composition of the Sixth National Population Census of China (2010). The 1999 World Health Organization (WHO) diagnostic criteria were used to diagnose Diabetes Mellitus [23]. A subject with SBP or DBP ≥140/90 mmHg is considered hypertension by WHO/ISH 1999 criteria [24].

MetS z-score was calculated. It takes into account continuous changes in each component, representing the score of continuous risk for MetS [25]. For each risk factor, a z-score was calculated (individual value - sample mean/standard deviation of the sample). For the blood pressure, we used the MAP (2/3 DBP + 1/3 SBP) of for calculating the score. Total score = waist Z score + BP Z score + glucose Z score + HDL-C Z score + triglycerides Z score. A lower risk score is indicative of a better metabolic profile.

### Statistical analysis

All analyses were stratified by sex. We grouped participants by age as follows: 18-45 yrs. (*n* = 1044), 46-55 yrs. (*n* = 1088), 56-65 yrs. (*n* = 1130) and > 65 yrs. (*n* = 839). Continuous variables are presented as means (SD, normal distributions) and median (interquartile range, skewed distributions) and proportions were calculated for discrete variables. To test differences of characteristics among different age groups, the 1-way ANOVA was applied for continuous data (FBG and TG were normalized by inverse and logarithmic transformation) and the Pearson chi-square test with linear by linear association was performed for categorical variables. Univariate and multivariable logistic regression analysis was performed to determine odds ratios (ORs) and 95% confidence intervals (CIs) for TG disorder and conventional risk factors. In the process of analyzing the frequency of components combination of MetS, all kinds of 3-factor combinations were counted. The actual frequency of one 3-factor combination is equal to the sum of the number of this combination in 3-factor group and the number of 4-factor and 5-factor group including this 3-factor combination (The theoretical frequency of total random 3 parameters combination equal to (total 3-factor frequency)*1 plus (total 4-factor frequency)*4 plus (total 5-factor frequency)*5). Data were analyzed using SPSS software version 22.0 for Mac, with the significance level set at *p* < 0.05 for all analyses.

## Results

A total of 9969 subjects (females, 5868) with a mean ± SD age of 53.34 ± 12.99 years were analyzed in this study. All participants were of Han origin. From Table 1, compared with males, the female participants had lower educational level, more manual worker, less current tobacco smokers, less alcohol drinkers and less recognized hypertensive subjects. The living area, marital status, history of DM and CVD were not statistically different between men and women. The crude MetS prevalence, using the IDF/AHA harmonized criteria, were significantly higher in females than males (32.3% vs 28.5%, *P* < 0.001). However, the age-standardized MetS prevalence showed no difference between genders (21.9% vs 22.0%, *P* > 0.05).

**Table 1:** Characteristics of study population by gender

	Total (*n* = 9969)	Male (*n* = 4101)	Female (*n* = 5868)	*P* value
Age (year)	53.34(12.99)	54.05(13.09)	52.85(12.90)	< 0.001
BMI (kg/m2)	24.53(3.52)	24.86(3.35)	24.30(3.62)	< 0.001
Living area, %				> 0.05
Rural	58.4	59.1	57.8	
Urban	41.6	40.9	42.2	
Educational level, %				< 0.001
High school or above	20.6	25.8	16.9	
Middle school	44.1	47.1	42	
Primary school or below	35.3	27.1	41.1	
Marital status, %				> 0.05
Married/co-habiting	93.8	93.9	93.8	
Single/divorced/widowed	6.2	6.1	6.2	
Occupational position, %				< 0.001
Manual	52.7	49.1	55.2	
Non-manual	34.8	35.5	34.2	
Self-employed	12.6	15.4	10.6	
Current smoking status, %	21.2	48.2	2.3	< 0.001
Current drinking status, %	54.1	68.1	44.3	< 0.001
History of hypertension, %	26.1	28.7	24.3	< 0.001
History of DM, %	7.3	7.6	7.1	> 0.05
History of CVD, %	5.7	5.3	6.1	> 0.05
Metabolic syndrome, % (unstandardized)	30.8	28.5	32.3	< 0.001
Metabolic syndrome, % (standardized)	22.0	21.9	22.0	> 0.05

Data were showed as means ± SD or percentages (%). *BMI* body mass index, *DM* diabetes mellitus, *CVD* Cardiovascular diseases

**Fig. 1:**
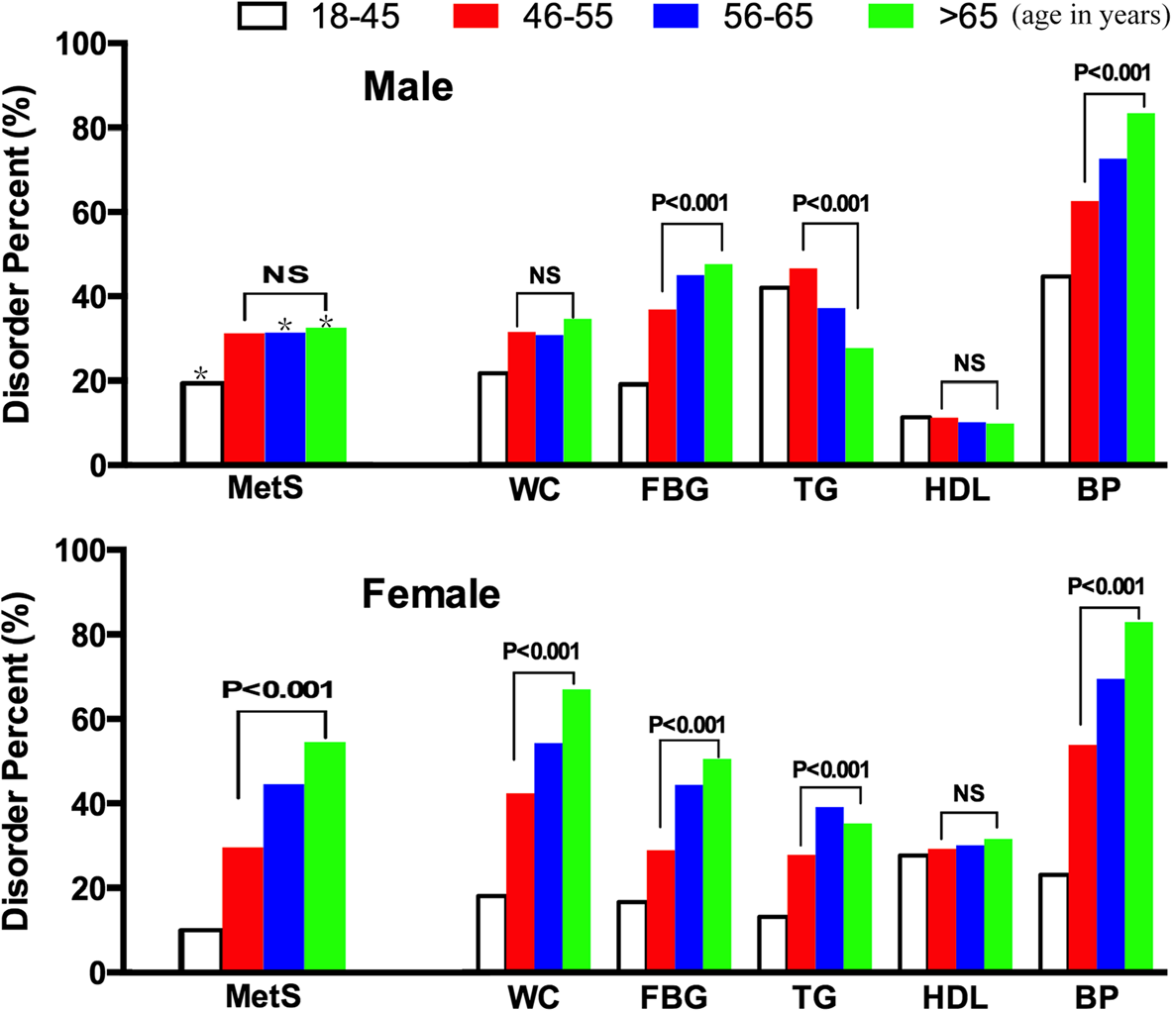
Frequency of MetS and each MetS component according to gender and age group. *, *P* < 0.001, compare male with female MetS from same age group. *P* value: linear by linear association of Chi-Square Tests among 46–55, 56–65 and > 65 yrs. age group. NS indicates non-significance

The prevalence of MetS and its components between age groups (18–45, 46–55, 56–65, > 65 yrs) were shown in Fig. 1. In female subjects, the frequency of MetS rose rapidly with age increase (10.4%, 29.9%, 44.9%, 54.9%, respectively, *P* < 0.001). In males, although the MetS frequency was significantly higher in older subjects (> 45 yrs) than 18-45 yrs. age group, there were no differences among 46–55, 56–65 and > 65 yrs. age groups, which meant the MetS prevalence did not go up with age in males. In 18-45 yrs. age group, the frequency of MetS was higher in males than females (19.4% vs 10.4%, *P* < 0.001). In older 56–65 and > 65 yrs. age groups, the frequency of MetS became lower in males than female counterparts (31.4% vs 44.9%, 32.5% vs 54.9%, both *P* < 0.001). To explore the underlying factors influencing MetS prevalence, the frequency of MetS component abnormalities (among 46–55, 56–65, > 65 yrs. age groups) were analyzed. In females, all of the MetS components showed increased abnormal frequencies with age except HDL, which kept stable across different age groups. In males, the FBG and BP abnormalities displayed similar ]trend as that in females and the WC and HDL abnormalities kept stable. However, the abnormal TG frequencies decreased with age (46.6%, 37.2%, 27.7%, respectively, *P* < 0.001). The values of MetS components showed similar trends as their abnormal frequencies (Table 2). These results suggest the non-increase of male MetS prevalence with age could be attributed to the improved TG disorders.

**Table 2:** The values of MetS components by age in both genders

	Male					Female				
	18–45 (*n* = 1044)	46–55 (*n* = 1088)	56–65 (*n* = 1130)	> 65 (*n* = 839)	*P* value	18–45 (*n* = 1748)	46–55 (*n* = 1540)	56–65 (*n* = 1592)	> 65 (*n* = 988)	*P* value
WC (cm)	82(76,88)	85(79,91)	84(78,91)	86(78,92)	0.485	71(66,77)	78(71,84)	80(74,87)	83(77,90)	< 0.001
FBG (mmol/L)	5.10(4.74,5.44)	5.35(4.96,5.91)	5.50(5.05,6.11)	5.54(5.10,6.20)	0.123	5.05(4.75,5.38)	5.22(4.89,5.70)	5.46(5.04,6.08)	5.56(5.11,6.31)	< 0.001
TG(mmol/L)	1.495(1.03,2.27)	1.595(1.12,2.44)	1.39(0.99,2.05)	1.22(0.89,1.78)	< 0.001	0.96(0.73,1.32)	1.27(0.92,1.76)	1.48(1.07,2.08)	1.42(1.09,1.95)	0.014
HDL (mmol/L)	1.27(1.11,1.46)	1.30(1.12,1.51)	1.35(1.16,1.56)	1.37(1.15,1.59)	< 0.001	1.45(1.28,1.67)	1.46(1.26,1.67)	1.45(1.26,1.66)	1.43(1.24,1.66)	0.26
SBP (mmHg)	124(114,134)	130(119,145)	136(124,151)	143(130,159)	< 0.001	115(106,125)	128(116,142)	136(123,150)	145(131,159)	< 0.001
DBP (mmHg)	79(70,87)	84(75,93)	83(75,92)	82(73,90)	< 0.001	72(65,79)	78(70,88)	80(71,87)	80(72,88)	0.082

Data were showed as median (interquartile range). *P* value: Linear Term of ANOVA among 46–55, 56–65 and > 65 yrs. age groups

We conducted a multiple logistic regression analysis of TG disorders and its associated factors in subjects aged older than 45 yrs. (Table 3, factors information see Additional file 1: Table S1). In males, individuals who have higher educational level or higher BMI were more likely to have TG disorders. Current smoking status, aged 56 yrs. or older were found to be inversely associated with having TG disorders. In females, aged 56 yrs. or older, higher BMI and manual occupation were associated with higher TG disorders.

**Table 3:** Associated factors of TG disorders in multivariate logistic regression analysis

	Male		Female	
Variable	Odds ratio (95%CI)	*P* value	Odds ratio (95%CI)	*P* value
Age (years)				
46–55	1.0		1.0	
56–65	0.798(0.651,0.979)	0.03	1.568(1.306,1.882)	< 0.001
> 65	0.632(0.499,0.800)	< 0.001	1.413(1.140,1.751)	0.002
BMI (kg/m2)	1.2(1.167,1.234)	< 0.001	1.146(1.121,1.171)	< 0.001
Living area				
Urban	1.0		1.0	
Rural	1.069(0.865,1.321)	0.535	1.007(0.846,1.197)	0.939
Educational level				
Primary school or below	1.0		1.0	
Middle school	1.252(1.009,1.552)	0.041	1.006(0.832,1.216)	0.952
High school or above	1.566(1.118,2.194)	0.009	1.041(0.727,1.490)	0.827
Marital status				
Married/co-habiting	1.0		1.0	
Single/divorced/widowed	1.59(0.947,2.668)	0.079	1.019(0.743,1.399)	0.905
Occupational position				
Manual	1.0		1.0	
Non-manual	0.893(0.670,1.189)	0.438	0.676(0.491,0.930)	0.016
Self-employed	0.871(0.688,1.103)	0.253	0.896(0.732,1.097)	0.289
Current smoking status				
Yes	1.0		1.0	
No	1.192(1.003,1.416)	0.046	1.065(0.669,1.695)	0.79
Current drinking status				
Yes	1.0		1.0	
No	1.069(0.883,1.293)	0.495	1.135(0.974,1.322)	0.105

In view of the opposite alteration of BP,BG and TG disorders with age increase in males, we further analyzed the change of metabolic disorder spectrum in the MetS population among age groups. From Table 4, in male MetS, the frequency of TG disorder was getting less and BP disorder was getting more with older age. The frequency of abnormal components changed from (TG > BP > WC > FBG > HDL) to (BP > FBG > WC > TG > HDL). In female MetS subjects, the markedly increased frequency of abnormal FBG and BP, together with decreased rate of abnormal TG and HDL with age, also dramatically changed the metabolic disorder spectrum. To quantify the total impact of metabolic disorders, we calculated the MetS z-score and found that the whole metabolic profile got better with older age in males (56-65 yrs. vs 46-55 yrs., > 65 yrs. vs 46-55 yrs., both *P* < 0.05), but in females the whole metabolic profile showed worse change (56-65 yrs. vs 46-55 yrs., *P* < 0.05).

**Table 4:** The frequency of abnormal components in MetS subjects

	Male MetS subjects					Female MetS subjects				
Disorders	18–45 (*n* = 203)	46–55 (*n* = 339)	56–65 (*n* = 355)	> 65 (*n* = 273)	*P* value	18–45 (*n* = 181)	46–55 (*n* = 460)	56–65 (*n* = 715)	> 65 (*n* = 542)	*P* value
WC, n (%)	135(66.5)	242(71.4)	246(69.3)	193(70.7)	0.828	138(76.2)	387(84.1)	604(84.5)	467(86.2)	0.61
FBG, n (%)	109(53.7)	242(71.4)	271(76.3)	206(75.5)	0.29	90(49.7)	264(57.4)	464(64.9)	380(70.1)	< 0.001
TG, n (%)	192(94.6)	297(87.6)	274(77.2)	182(66.7)	< 0.001	116(64.1)	300(65.2)	495(69.2)	310(57.2)	< 0.001
HDL, n (%)	67(33.0)	85(25.1)	89(25.1)	66(24.2)	0.959	144(79.6)	288(62.6)	386(54.0)	282(52.0)	0.002
BP, n (%)	182(89.7)	298(87.9)	329(92.7)	266(97.4)	< 0.001	136(75.1)	384(83.5)	632(88.4)	521(96.1)	< 0.001
Z-score	1.40(−0.05,3.27)	1.31(−0.00,3.63)	1.11(−0.37,2.85) ^a^	0.88(−0.29,2.60) ^#^		0.96(−0.15,2.81)	1.35(0.08,3.34)	1.70(0.22,3.60)	2.03(0.58,3.80)^a^	

Data were showed as number of subjects (%) and median (interquartile range). *P* value: Chi-Square Tests among age 46–55, 56–65 and > 65 yrs. ^a^compare with 46-55 yrs. age group, *P* < 0.05; ^#^compare with 46-55 yrs. age group, *P* < 0.01

**Fig. 2:**
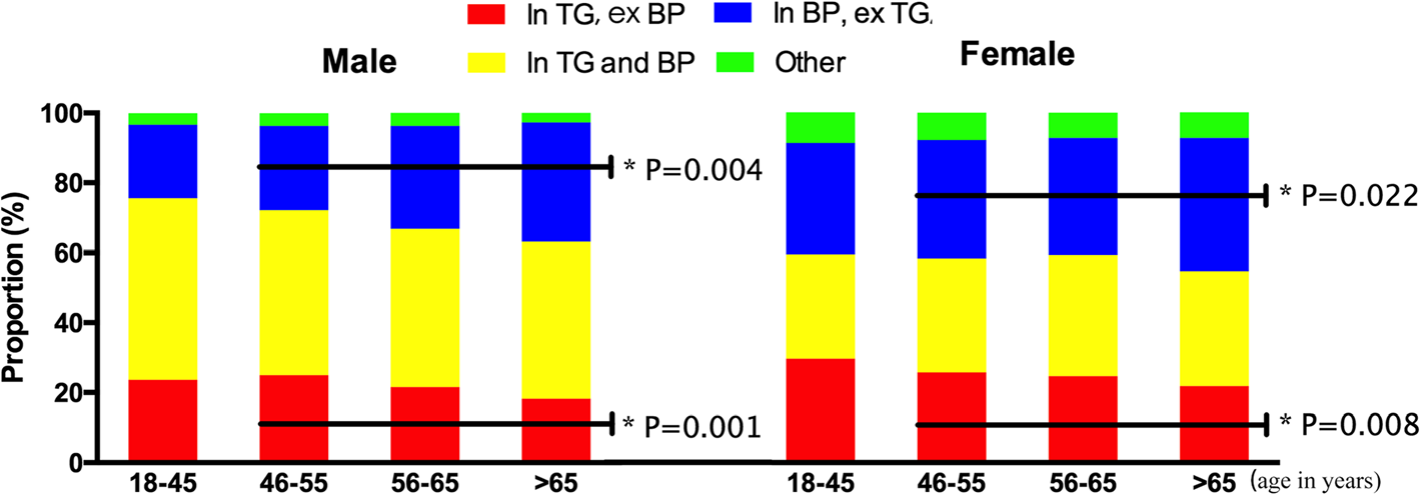
Comparison of various components clustering in all MetS subjects according to gender and age group. In: include, ex: exclude. *P* value: linear by linear association of Chi-Square tests among 46–55, 56–65 and > 65 yrs. age group

The clustering of various components was further assessed (Additional file 2: Table S2). Using 3 parameters as a combination, in males, we found the combinations which included BP and excluded TG (WC + BP + HDL, WC + BP + FBG, HDL + BP + FBG) accounted for more proportion in 56–65 and > 65 than 46-55 yrs. age group (29.5%, 34.2% vs 24.1%, both *P* < 0.05). On the contrary, the combinations which included TG and excluded BP (WC + TG + HDL, WC + TG + FBG, HDL + TG + FBG) took less proportion in 56–65 and > 65 than 46-55 yrs. age group (21.5%, 18.2% vs 25.0%, both *P* < 0.05). In females the similar trend did exist, but to a less extent (Fig. 2).

## Discussion

In this study, we investigated the age-related MetS prevalence between genders from a cross-sectional survey in East China. The results showed that unlike the continuous MetS rise with age in females, the MetS prevalence in males remained stable among aged 46 yrs. or older age groups, which could be attributed to the improved TG disorders. In the MetS subjects, the frequency of abnormal components also markedly changed, in which TG disorder became less frequent and BP disorder became more frequent with advancing age. The whole metabolic profile got better in older male MetS subjects, which was opposite to the female. To the best of our knowledge this is the first study to specifically describe the different pattern of MetS prevalence with age between genders and note the important role of TG played in this course in China. Our results provided a better understanding of the current characteristics of MetS in China for future specific prevention and treatment.

The MetS incidence kept stable in aged 46 yrs. or older age groups. This pattern was distinct from other population. From NHANES 1999–2000, the prevalence of MetS continued to rise among 20–39, 40–59 and ≥ 60 yrs. age groups. Another analysis investigated prevalence by decade of age from NHANES 2003–2006 and found the prevalence rose until aged 60–69 yrs. in both genders, and declined thereafter [26–28]. Data from 10 European countries also showed the MetS prevalence increased with advancing age (from 3.7 in the group aged 20–29 yrs. to more than 30% in the subjects 70 yrs. and older) [29, 30]. In a study from Saudi population, the prevalence of MetS increased uniformly with advancing age until aged 60–65 yrs. in both male and female [31]. However, a couple of previous studies from China displayed similar prevalence pattern to ours. Results from residents living in Northeast China rural area [17] showed the MetS prevalence kept stable among 35–44, 45–54 and 55-64 yrs. age groups. Another study from Northeast China even showed declining prevalence among 45–54, 55–64 and ≥ 65 yrs. age groups in males [19]. Above evidence suggest there might be a specific age-related prevalence of MetS in current Chinese population.

In the five components of MetS, the frequency of abnormal FBG, BP, WC and HDL increased or remained stable in older age groups in both genders. Abnormal TG is less frequent with advancing age in males, while more frequent in females, which suggest the opposite TG changes play a key role in the gender-specific prevalence pattern. The decrease of TG with older age in males had ever been reported. An epidemiological survey in China found the percentage of abnormal TG was lower in ≥60 yrs. than in 44-59 yrs. in males [32]. The MORGAM research in European subjects showed the same abnormal TG trend as that in our study in both genders among 40–49, 50–59 and 60-78 yrs. age groups [33]. However, in NHANES (2003–2006) and Framingham Offspring Study [26, 27, 34], the TG disorders continued to rise until aged 60–69 yrs. in both genders. The exact mechanisms regarding age-related TG change between genders have not been clarified yet. Various mechanisms have been proposed to explain the differences. There is an obvious difference in fat distribution between men and women [35]. One study has suggested that the redistribution of body fat is associated with the TG change in older women [36]. Other evidence suggests that in postmenopausal and premenopausal women, the metabolic changes might be attributed to the different hormonal levels, with the sex hormones being of major importance [37]. In our population, the molecular mechanisms of TG change with age and the role of sex hormone in this process need more laboratory work.

Another thing to be noted is that the average TG levels showed decline tendency recently in some countries, e.g. the US [38, 39], while in China the average TG levels is still increasing [40, 41]. We think the decrease of TG in males does not just come from the age, but also reflect differences between the subjects. In the current study, the incidence of male TG disorders were positively associated with higher educational level or higher BMI and negatively associated with current smoking status. Socioeconomic status has long been known to predict higher rates of many chronic diseases and low socioeconomic status may affect women’s health to a greater extent [42, 43]. These sociodemographic characteristics may contribute to the decline of TG in older age groups and people with these risk factors should be the important target population of prevention and control of MetS.

Although the prevalence remained stable across age groups, the metabolic disorder spectrum dramatically changed in male MetS subjects, which characterized by less frequent TG disorders and more frequent BP disorders in older age groups. The trends of abnormal BP and BG were noted in several Chinese studies [44] in which abnormal BP became the most common component. This was significantly different from western countries, where abnormal WC remained the most common component [27, 45] and BP disorder even decreased [46].

One interesting finding in our analysis was the whole metabolic profile got better in male older age groups. This meant the total disorder degree of five components of MetS, which were equally weighted in the calculation, improved with age. Although the clinical prognostic significance of z-score needs further investigation, at least from the value the five components of MetS got better controlled in older males.

The age-standardized prevalence of metabolic syndrome reported in our study was 21.9% in men and 22.0% in women, and there was no statistical difference between genders. Adopting the same diagnostic criteria, the non-standardized prevalence 33.9% (31.0% in men and 36.8% in women) from the 2010 China Non-communicable Disease Surveillance [20] and 39% (31.4% in men and 45.6% in women) from Rural China obviously overestimated the MetS prevalence in China [17]. Actually, the overall age-standardized prevalence from the China Health and Nutrition Survey conducted in 2009 were 21.3% (20.9% in males and 21.7% in females) [16], which was quite close to our results. The standardized prevalence of MetS was even lower in rural area of Northwest China (15.1%, 12.8% in males and 17.4% in females) [18]. We think the overall MetS prevalence of Chinese is lower than the US American (34.3%) [27], but comparable to Canadians (19.1%) [45] and other Asian countries [47]. As to gender difference of prevalence, in our study, the higher percentage at early age and lower percentage at old age in males make the overall prevalence equal between genders. Data from the NHANES [28] and the Canadian [45] studies also showed no differences in the overall prevalence of MetS between genders. Whether the similar prevalence between genders is just a transient balance or there are other unknown mechanisms to the equilibrium needs further investigation.

Over the last few decades, China’s remarkable economic development has significantly improved people’s living standards, including food consumption and nutritional status. The per-capita consumption of proteins steadily increased from 1989 to 2009, especially the proteins from dairy products and processed meat. The intake of oils and fats also increased, but consumption of carbohydrates remained more or less stable. Moreover, fruit and vegetable intake increased significantly over that period [48]. Previous studies have found that excessive caloric intake, processed meat and fried foods could promote the incidence of MetS [49]. However, a high consumption of fruit, vegetables and dairy products is associated with a decreased risk of having MetS. Greater intakes of fruit and vegetables also are associated with lower triglycerides [50]. In our study, gender differences in dietary intake among Chinese adults and their role in the incidence of MetS require further exploration.

There were several limitations in the present study. First, our cross-sectional study only gave information about the prevalence among age groups, not with aging. Therefore, future studies, especially prospective studies are needed to confirm the prevalence difference with aging. Second, the prevalence of MetS and its components would change over time and the diagnostic criteria of MetS were not uniform before 2010, the comparisons between the prevalence need to be explained cautiously. Third, the number of very old (> 75 yrs) people in our participants was small and they were not divided as another age group. Some previous research showed declined prevalence of MetS in this age group [26], so our results for the oldest age group (≥65 yrs) should be interpreted with caution. Fourth, in the logistic analysis of TG disorders, we only collected and analyzed participants’ occupation, marital status and educational level. Some important risk factors, such as family income, diet behaviors and physical activity data are not taking into account, so the associations of these factors with TG disorders are not clear.

## Conclusions

In summary, unlike the continuous MetS rise with age in females, the MetS prevalence in males was higher than that in females in 18-45 yrs. age group and remained stable among aged 46 yrs. or older age groups, which could be attributed to the improved TG disorders. The less frequent TG disorder and more frequent BP disorder in old Chinese MetS subjects need more targeted intervention strategies to be carried out to meet the serious challenges of metabolic diseases.

## Additional files

Additional file 1: **Table S1.** The sociodemographic characteristics by age in both genders (DOCX 22 kb)

Additional file 2: **Table S2.** The clustering of various components by age and gender (DOCX 22 kb)

## Abbreviations

BMI: Body mass index
BP: Blood pressure
CVD: Cardiovascular disease
DM: Diabetes mellitus
FBG: Fasting blood glucose
HDL-C: High-density lipoprotein cholesterol
MetS: Metabolic syndrome
TG: High triglyceride
WC: Waist circumference
