# Supplementary material for: Age and gender-specific distribution of metabolic syndrome components in East China: role of hypertriglyceridemia in the SPECT-China study
Source: Lipids Health Dis. 2018 Apr 20;17:92. doi: 10.1186/s12944-018-0747-z (PMC5910574; doi:10.1186/s12944-018-0747-z)
Supplement: Supplementary file 1 — Table S1. The sociodemographic characteristics by age in both genders (DOCX 22 kb) [file 12944_2018_747_MOESM1_ESM.docx]

Additional file 1: **Table S1** The sociodemographic characteristics by age in both genders

|  | Male | | | |  | Female | | | |  |
| --- | --- | --- | --- | --- | --- | --- | --- | --- | --- | --- |
|  | 18-45  (n=1044) | 46-55  (n=1088) | 56-65  (n=1130) | >65  (n=839) | P value | 18-45  (n=1748) | 46-55  (n=1540) | 56-65  (n=1592) | >65  (n=988) | P value |
| **Living area (%)** |  |  |  |  |  |  |  |  |  |  |
| Rural | 50.40% | 55.50% | 61.90% | 70.90% | <0.001 | 50.20% | 60.60% | 60.90% | 62.00% | <0.001 |
| Urban | 49.60% | 44.50% | 38.10% | 29.10% |  | 49.80% | 39.40% | 39.10% | 38.00% |  |
| **Educational level (%)** |  |  |  |  |  |  |  |  |  |  |
| High school or above | 53.80% | 28.00% | 10.30% | 10.20% | <0.001 | 40.20% | 12.70% | 4.00% | 4.50% | <0.001 |
| Middle school | 42.20% | 57.10% | 50.40% | 35.60% |  | 49.50% | 58.10% | 33.50% | 18.30% |  |
| Primary school or below | 4.00% | 14.90% | 39.30% | 54.30% |  | 10.30% | 29.20% | 62.60% | 77.20% |  |
| **Marital status (%)** |  |  |  |  |  |  |  |  |  |  |
| Single/divorced/widowed | 13.00% | 1.90% | 3.00% | 6.80% | <0.001 | 7.50% | 2.80% | 4.40% | 12.20% | <0.001 |
| Married/co-habiting | 87.00% | 98.10% | 97.00% | 93.20% |  | 92.50% | 97.20% | 95.60% | 87.80% |  |
| **Occupational position (%)** |  |  |  |  |  |  |  |  |  |  |
| Manual | 35.00% | 43.40% | 54.80% | 66.10% | <0.001 | 36.30% | 54.60% | 64.90% | 73.70% | <0.001 |
| Non-manual | 41.60% | 40.10% | 30.80% | 28.60% |  | 46.50% | 33.80% | 27.90% | 23.50% |  |
| Self-employed | 23.40% | 16.40% | 14.50% | 5.30% |  | 17.20% | 11.60% | 7.20% | 2.80% |  |
| **Current smoking status (%)** |  |  |  |  | <0.001 |  |  |  |  | <0.001 |
| Yes | 40.60% | 54.30% | 52.10% | 44.60% |  | 1.40% | 0.90% | 2.60% | 5.80% |  |
| **Current drinking status (%)** |  |  |  |  | <0.001 |  |  |  |  | <0.001 |
| Yes | 63.60% | 75.10% | 68.80% | 63.50% |  | 37.60% | 49.10% | 44.70% | 48.40% |  |

Values are expressed as percentages. Chi-square test or Fisher exact test are performed to compare percentages among age groups.
