# Supplementary material for: Age and gender-specific distribution of metabolic syndrome components in East China: role of hypertriglyceridemia in the SPECT-China study
Source: Lipids Health Dis. 2018 Apr 20;17:92. doi: 10.1186/s12944-018-0747-z (PMC5910574; doi:10.1186/s12944-018-0747-z)
Supplement: Supplementary file 2 — Table S2. The clustering of various components by age and gender (DOCX 22 kb) [file 12944_2018_747_MOESM2_ESM.docx]

|  |  | Male | | | | Female | | | |
| --- | --- | --- | --- | --- | --- | --- | --- | --- | --- |
|  |  | 18-45 | 46-55 | 56-65 | >65 | 18-45 | 46-55 | 56-65 | >65 |
|  |  | n=1044 | n=1088 | n=1130 | n=839 | n=1748 | n=1540 | n=1592 | n=988 |
| 0 factor |  | 312 | 163 | 117 | 54 | 764 | 285 | 122 | 40 |
|  |  |  |  |  |  |  |  |  |  |
| 1 factor | WC | 28 | 20 | 20 | 15 | 56 | 80 | 53 | 22 |
|  | FBG | 32 | 49 | 63 | 28 | 102 | 59 | 59 | 22 |
|  | BP | 129 | 149 | 194 | 175 | 130 | 176 | 152 | 79 |
|  | TG | 91 | 50 | 34 | 12 | 30 | 38 | 25 | 4 |
|  | HDL | 12 | 12 | 2 | 3 | 189 | 54 | 24 | 6 |
|  |  |  |  |  |  |  |  |  |  |
| 2 factor | WC+FBG | 4 | 3 | 6 | 5 | 22 | 22 | 38 | 30 |
|  | WC+BP | 39 | 55 | 63 | 73 | 39 | 115 | 133 | 126 |
|  | WC+TG | 19 | 19 | 10 | 3 | 18 | 16 | 21 | 10 |
|  | WC+HDL | 2 | 4 | 3 | 1 | 43 | 33 | 15 | 7 |
|  | FBG+BP | 26 | 75 | 145 | 151 | 37 | 68 | 113 | 62 |
|  | FBG+TG | 28 | 32 | 20 | 7 | 9 | 17 | 19 | 4 |
|  | FBG+HDL |  | 1 | 3 | 2 | 32 | 15 | 14 | 1 |
|  | BP+TG | 81 | 96 | 77 | 26 | 22 | 42 | 50 | 18 |
|  | BP+HDL | 10 | 8 | 13 | 9 | 38 | 45 | 26 | 13 |
|  | TG+HDL | 28 | 13 | 5 | 2 | 36 | 15 | 13 | 2 |
|  |  |  |  |  |  |  |  |  |  |
| 3 factor | WC+FBG+BP | 5 | 29 | 57 | 64 | 13 | 54 | 99 | 113 |
|  | WC+FBG+TG | 8 | 20 | 12 | 5 | 3 | 12 | 12 | 7 |
|  | WC+FBG+HDL |  | 2 | 1 |  | 7 | 12 | 11 | 5 |
|  | WC+BP+TG | 53 | 64 | 54 | 34 | 11 | 50 | 85 | 48 |
|  | WC+BP+HDL | 3 | 4 | 5 | 12 | 26 | 47 | 42 | 54 |
|  | WC+TG+HDL | 7 | 6 | 4 | 2 | 18 | 27 | 23 | 4 |
|  | FBG+BP+TG | 38 | 66 | 70 | 50 | 5 | 18 | 26 | 22 |
|  | FBG+BP+HDL | 2 | 4 | 9 | 10 | 12 | 18 | 17 | 17 |
|  | FBG+TG+HDL | 4 | 6 | 6 |  | 5 | 8 | 17 |  |
|  | BP+TG+HDL | 20 | 10 | 11 | 13 | 15 | 17 | 31 | 15 |
|  |  |  |  |  |  |  |  |  |  |
| 4 factor | WC+FBG+BP+TG | 32 | 75 | 73 | 54 | 5 | 38 | 107 | 71 |
|  | WC+FBG+BP+HDL | 1 | 3 | 9 | 5 | 7 | 29 | 51 | 43 |
|  | WC+BP+TG+HDL | 11 | 13 | 10 | 6 | 21 | 55 | 70 | 41 |
|  | WC+FBG+TG+HDL | 2 | 7 | 3 |  | 12 | 17 | 20 | 4 |
|  | FBG+BP+TG+HDL | 4 | 11 | 13 | 7 | 6 | 12 | 20 | 21 |
| 5 factor |  | 13 | 19 | 18 | 11 | 15 | 46 | 84 | 77 |

Additional file 2: **Table S2** The clustering of various components by age and gender

Data were showed as number of subjects (n).
